# Biofilm formation on venovenous ECMO cannulas can lead to re-introduction of pathogens during the decannulation process – a small-scale study reveals new insights when combining cultures and molecular results

**DOI:** 10.1186/s12879-026-12731-x

**Published:** 2026-02-05

**Authors:** Simone Kattner, Marcel Hochreiter, Ann-Kathrin Dörr, Andrea Engler, Hannah Möhlen, Verena Freitag, Ksenia Pawlytta, Thorsten Brenner, Folker Meyer, Ivana Kraiselburd

**Affiliations:** 1 Department of Anesthesiology and Intensive Care Medicine, University Hospital Essen, University Duisburg-Essen, Essen, Germany; 2 Institute for AI in Medicine, University Hospital Essen, University of Duisburg-Essen, Essen, Germany; 3 Department of Anesthesiology and Intensive Care Medicine, Westcoast Hospital, Heide, Germany

**Keywords:** 16S rDNA, Sepsis, ECMO, Acute respiratory distress syndrome

## Abstract

**Background:**

Extracorporeal membrane oxygenation (ECMO) cannulas are potential reservoirs for pathogens, yet their role in bacteremia and sepsis following decannulation remains poorly understood. This proof-of-concept study aims to characterize bacterial colonization of ECMO cannulas, identify potential sources of these bacteria, and assess their association with post-decannulation bloodstream infections and sepsis.

**Methods:**

We conducted a single-center observational study including 10 patients receiving venovenous ECMO support between January 2022 and January 2023. Microbial colonization of cannulas, skin sites, and plasma was analyzed using culture-based methods and 16S rDNA amplicon sequencing. Alpha and beta diversity analyses were performed, and findings were correlated with clinical outcomes, including sepsis and bacteremia.

**Results:**

A total of 117 samples yielded ~ 11 million sequencing reads. Bacteria colonizing ECMO cannulas matched pathogens causing prior bacteremia during ECMO support in all affected patients. Bacteria detected on cannulas and insertion sites were frequently recovered in plasma following decannulation. Notably, 16S rDNA analysis detected circulating pathogens that conventional cultures missed, often those from prior infections that were thought to be eradicated by antibiotics. Patients who developed sepsis post-decannulation exhibited higher bacterial diversity on cannulas and a higher overall abundance of *Pseudomonas*, while non-septic patients had greater *Enterococcus* abundance.

**Conclusions:**

Our results confirm that ECMO cannulas serve as pathogen reservoirs, with decannulation enabling bacterial translocation into the bloodstream and contributing to post-decannulation sepsis. 16S rDNA sequencing exhibited greater sensitivity than cultures for detecting bloodstream pathogens. These findings support re-assessment of prophylactic measures during ECMO decannulation and lay the groundwork for developing early sepsis risk stratification tools.

**Supplementary Information:**

The online version contains supplementary material available at 10.1186/s12879-026-12731-x.

## Background

ECMO (extracorporeal membrane oxygenation) is an artificial life support system for patients with severe cardiac and/or pulmonary failure [1]. In 2024, the Extracorporeal Life Support Organization recorded approximately 63,876 ECMO treatments for lung failure and 66,402 for heart failure in adults worldwide [2]. Venovenous (VV) ECMO is commonly used in lung failure or acute respiratory distress syndrome (ARDS), while venoarterial (VA) ECMO is commonly applied for cardiovascular failure. While ECMO provides life-saving support in critical illness, it carries a substantial risk for nosocomial infections which can poorly influence ECMO survival and overall survival [3–7]. Both modalities of ECMO require insertion of large-bore cannulas in central vessels, where microbial biofilms can evolve [8, 9] and serve as potential reservoirs for secondary infections and antibiotic resistance. Bacteria colonizing ECMO cannulas may originate from primary infection sites or be introduced during cannulation [10]. The ECMO oxygenator may also act as a source of microorganisms [11, 12].

Blood cultures as well as cannula cultures, although representing the gold standard for pathogen diagnostics, are time-consuming and prone to false-positive and false-negative results [13–15]. Modern molecular methods may help to overcome these limitations. A widely used technique for characterizing prokaryotic communities is 16S rDNA amplicon sequencing. This involves PCR amplification of conserved regions of the small subunit ribosomal RNA gene [16, 17]. The method offers rapid turnaround, high sensitivity, and reliable taxonomic classification supported by robust analysis tools.

While a recent study found no correlation with the presence of biofilms in ECMO cannulas and patient outcomes using cultivation-based methods and quantitative measures of bacterial DNA in catheters [18], previous studies employing 16S rDNA sequencing to evaluate bacterial biofilms on ECMO cannulas, reported microbiome differences associated with bacteremia development [19]. These contradictory findings highlight a knowledge gap about the clinical significance of cannula-associated biofilms. To shed new light on this situation, we evaluate bacterial colonization of ECMO cannulas and its effect in patient outcomes after decannulation. We aim at both identifying the sources of bacteria colonizing the cannulas and assessing cannula removal as risk factor for secondary infections after ECMO termination.

We employ 16S rDNA amplicon sequencing to characterize microbial communities on ECMO cannulas, the patient’s skin and blood, and compare these findings with conventional blood cultures.

## Methods

### Study design and ECMO characteristics

This single-center study included patients admitted to the intensive care unit (ICU) of the Department of Anesthesiology and Intensive Care Medicine, University Hospital Essen for ECMO therapy between January 2022 and January 2023. The study protocol was approved by the local ethics committee (BO Nr. 20-9637). Ten patients were included in the final analysis. Demographic and clinical characteristics are summarized in Supplementary Table 1.

Eligible patients aged ≥ 18 years, received VV- or VA-ECMO for > 48 h, and provided informed consent. Pregnancy was an exclusion criterion.

The 10 patients in this study were supported with VV ECMO using Maquet HLS cannulas and the Maquet Cardiohelp System (HLS Module Advanced 7.0 oxygenator). Cannula sizing was adjusted per patient, with a draining venous cannula of at least 23 Fr and a venous cannula of at least 19 Fr for giving back blood.

The surviving patients were also part of a parallel study conducted under the same ethics approval [20]. Demographic data, microbiological results, and clinical outcomes were obtained retrospectively from medical records. The study design and reporting adhered to the STROBE guidelines [21].

### Definition of septic complications

Sepsis was defined according to the Sepsis-3 criteria as an increase in SOFA score of ≥ 2 points; septic shock was defined as SOFA Score increase of ≥ 2 points combined with lactate > 2 mmol/l [22]. Decannulation-related bacteremia was defined as at least one positive blood culture obtained after ECMO decannulation.

### Sampling and pre-processing

Before ECMO decannulation, blood was collected for cultures and 16S rDNA analysis via a newly placed sterile peripheral venous catheter, which was also used for a second sample within 10 min post-decannulation. Anticoagulated whole blood (9 mL EDTA) was centrifuged (10 min, 2000 g, 4 °C); 1 mL plasma was mixed with 1 mL phenol: chloroform: isoamyl alcohol, vortexed, and stored at − 80 °C until DNA extraction.

Dressings covering the cannulation sites were removed and patient’s skin (10 × 10 cm area) was swabbed 5 cm from the cannula site, outside the dressing area, wiping 20 times with cotton swabs pre-moistened with sterile TE buffer containing 0.5% Tween 20. The cannula insertion sites were swabbed in the same manner. Swab tips were stored at -80 °C until DNA extraction. After sampling, the cannulas insertion sites were disinfected (Kodan ^®^ Tinktur forte colored). Following decannulation, the insertion site was sutured, covered with compresses, and firm pressure was applied for 15 min. The compress was processed like the swabs.

The first 15 cm of each ECMO cannula were cut off with sterile scissors into 5 cm segments. Residual blood was carefully flushed out with up to 50 ml sterile 0.9% NaCl. For the first five patients, the 5 cm segments were sliced open, and the inner and outer surfaces were wiped with swabs pre-moistened with TE buffer. The cannula pieces were then placed in sterile tubes containing TE buffer for further processing using a vortex-sonication-vortex protocol following the European Society of Clinical Microbiology and Infectious Diseases (ESCMID) guidelines [23, 24]. Cannula pieces were centrifuged (200 x g, 10 min, 4 °C) to remove host cells. The supernatant and cannulas were transferred to a fresh falcon tube, vortexed (1 min), sonicated (1 min, 40 kHz) and vortexed again (1 min). After removal of the cannulas, the samples were centrifuged (4500 × g, 30 min, 4 °C) and the pellets stored at -80 °C until further processing.

Since no differences in taxonomic diversity were observed between swabbed and vortexed cannula samples, the protocol was adjusted for subsequent patients: The 15 cm cannula tips were placed directly into sterile tubes with TE buffer for proceeding to the vortex-sonication-vortex as described above. This approach improved sampling efficiency at the bedside.

### DNA extraction

DNA from swabs and cannula samples was extracted using the DNAeasy PowerSoil Kit (Qiagen), according to the manufacturer’s instructions. DNA from plasma samples was isolated using the phenol: chloroform: isoamino alcohol extraction (Thermo Fisher Scientific).

### 16S rDNA gene amplicon sequencing

Sequencing libraries were prepared following the 16S Metagenomic Sequencing Library Preparation protocol (Illumina), using the primers Bakt_341F and Bakt_805R targeting the V3-V4 region [25]. Commercial cell and DNA MOCK communities (ZymoBIOMICS) served as positive controls for the DNA extraction and library preparation. Libraries were normalized, pooled, denatured and spiked with 20% PhiX DNA. Sequencing was performed on an Illumina MiSeq instrument (V2 chemistry, 500 cycles).

### Data analysis

Sequencing data were processed using RiboSnake [26], including quality control, host read removal, and filtering (Phred ≥ 15, length ≥ 200 bp). Paired-end sequencing reads were merged, chimeras removed, and Operational Taxonomic Unit (OTUs) clustered with Vsearch [27, 28]. Taxonomic classification was performed using the SILVA SSU database release 138 [29]. To reduce false positives, reads were filtered for relative abundance ≥ 0.001, as described by Nearing et al. [30, 31]. Amplicon Sequence Variants (ASVs) were generated using DADA2 [32]. Samples from identical sites per patient were grouped using QIIME2’s “feature-table group” (mean-ceiling). Alpha/beta diversity and feature importance (Analysis of Composition of Microbiomes, ANCOM) were analyzed within RiboSnake [33, 34].

## Results

### 16S rDNA amplicon sequencing results

**Fig. 1:**
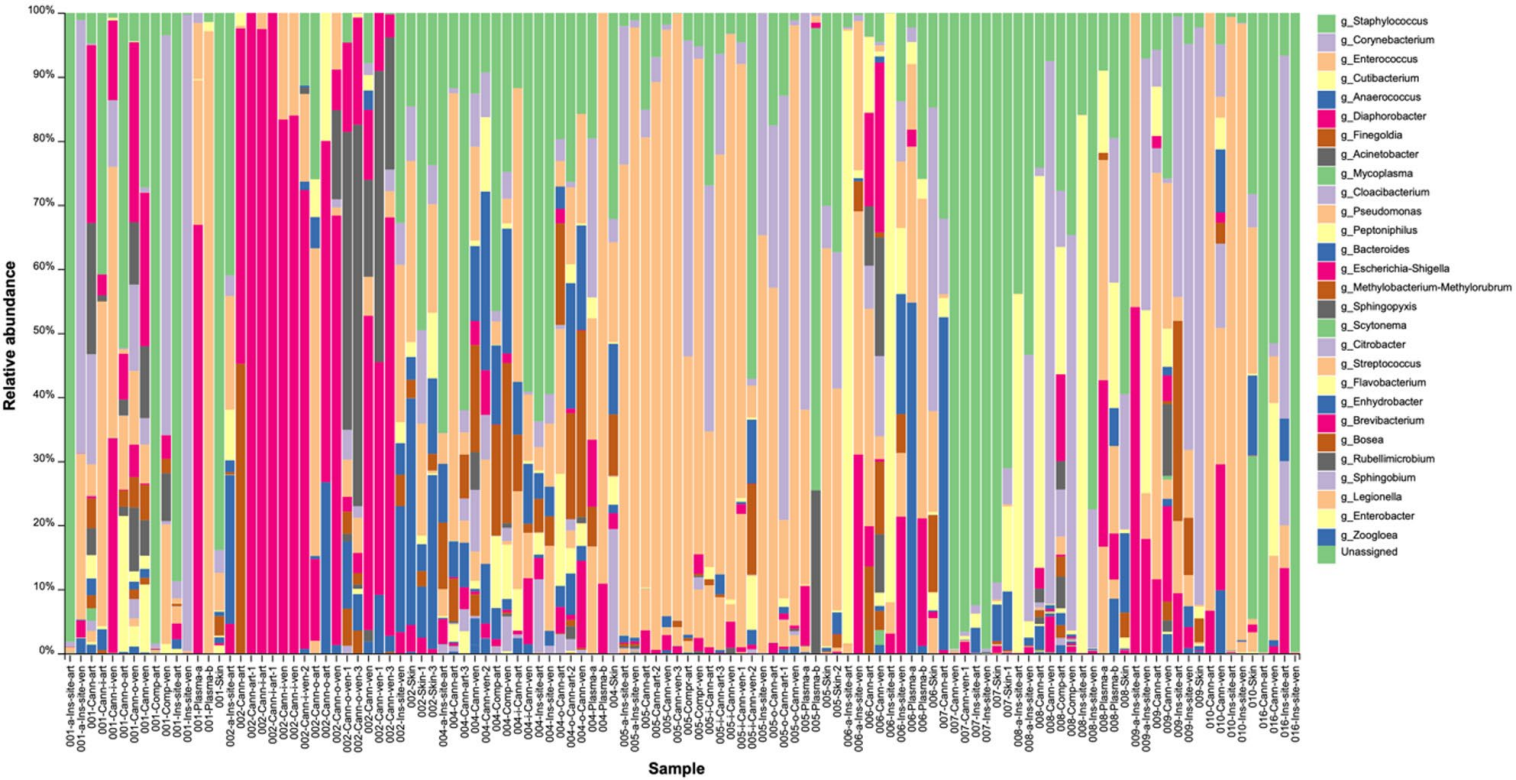
Bar plot displaying the relative abundance of genera identified across all samples, based on OTU (Operational Taxonomic Unit) data. Ins-site = cannula insertion site; Comp = compress; Cann= ECMO cannulas; a and b = before and after decannulation, respectively; ven = venous; art = arterial (both cannulas are venous, cannulas labeled as art refer to those giving back blood into a vein)

A total of 117 samples from five survivors and five non-survivors were sequenced, yielding ~ 11 million paired-end reads. Despite variations in total numbers of reads, sequencing depth was sufficient to capture the full diversity present in each sample, as confirmed by rarefaction analysis. Taxonomic classification of the OTUs allowed identification of a total of 28 taxa on the genus level observed in all samples with varying relative abundance (Fig. 1). Taxonomic analysis using ASVs instead of OTU yielded only marginal differences (data not shown).

**Fig. 2:**
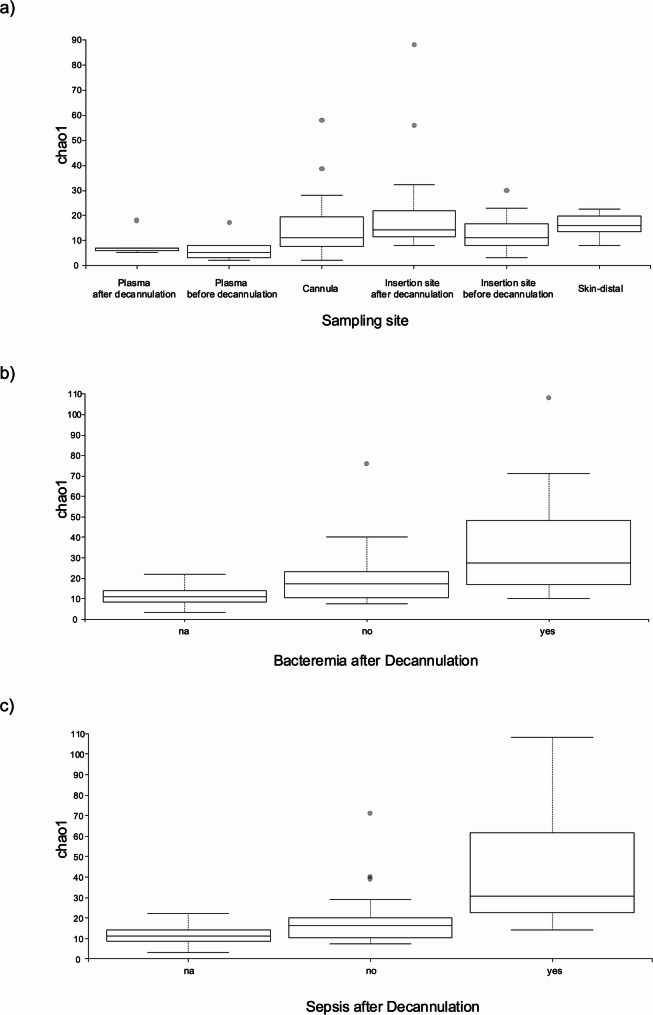
Alpha diversity (Chao1 index) from 16S rDNA data. (**a**) All samples by site and decannulation stage. (**b**–**c**) Cannula samples grouped by post-decannulation bacteremia (**b**) or sepsis (**c**)

Microbial diversity analyses of 16S rDNA data included alpha (“diversity of taxa within samples”) and beta (“changes in taxa across samples”) diversity metrics and feature importance assessment (“which taxa explain the differences between samples”). The aim was exploring microbial variation within and between samples and identifying potential biomarkers influencing post-decannulation outcomes. Alpha diversity (richness) values were the lowest in plasma samples (Fig. 2a, Supplementary Table 2a) followed by cannulas and insertion sites, with highest values on skin samples. Interestingly, alpha diversity for both plasma and insertion sites increased after decannulation. Richness increases were statistically significant at insertion sites but not in plasma. Nonetheless, this suggests bacterial release from cannulas into blood and surrounding tissue during decannulation. When evaluating alpha diversity exclusively in cannulas samples, higher values were observed in patients developing sepsis or bacteremia during a 7-days observational period after decannulation in comparison to those who did not (Fig. 2b-c, Supplementary Table 2b). The difference was only statistically significant for the comparison based on sepsis (pairwise Kruskal-Wallis p-value = 0.029).

**Fig. 3:**
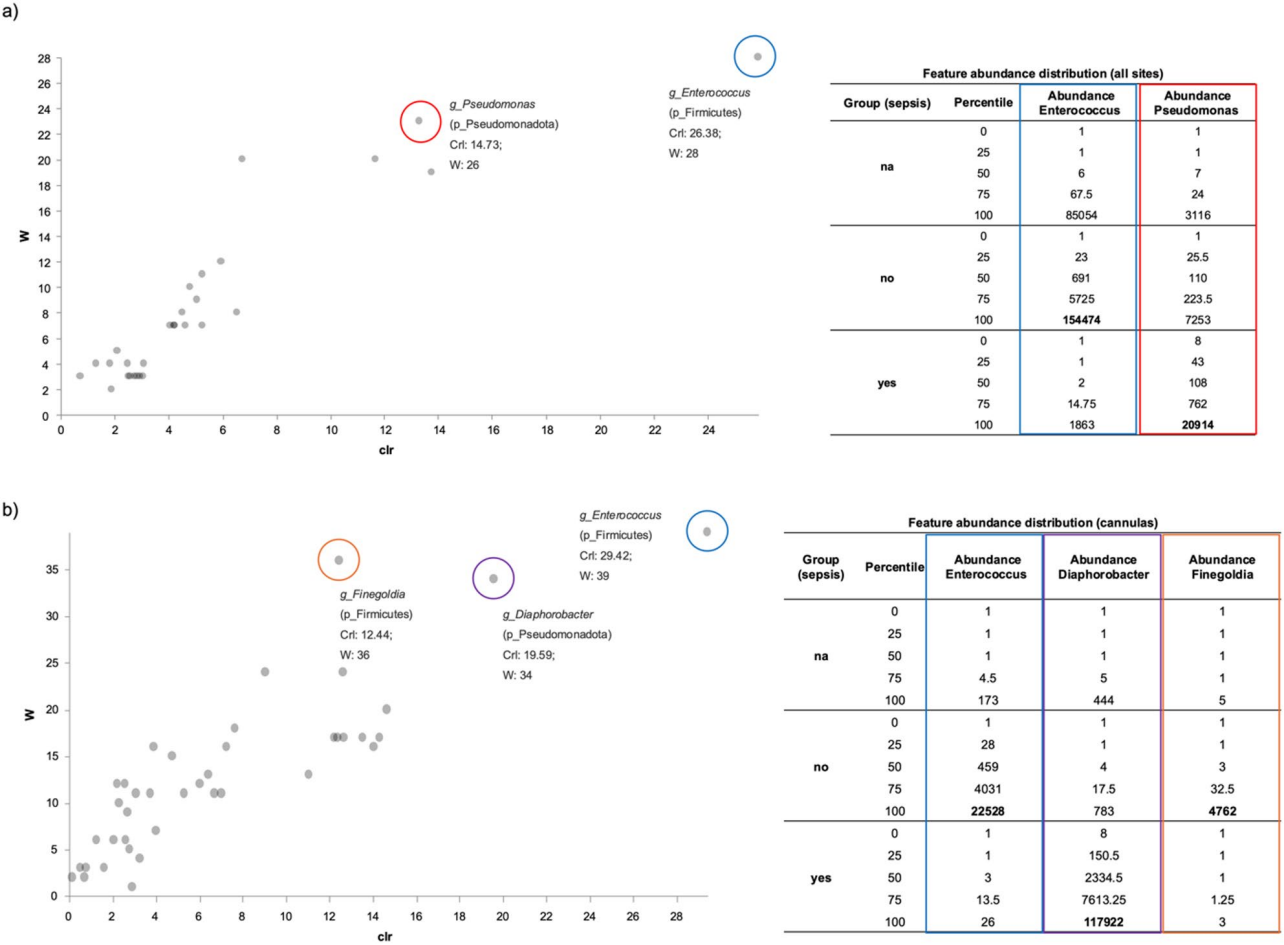
Analysis of Composition of Microbiomes (ANCOM) analysis of 16S rDNA sequencing data comparing patients with and without post-decannulation sepsis, for all sample types (**a**) and cannula samples only (**b**). Scatter plots show microbial genera with centered log-ratio (crl) abundance (x-axis) and W-statistics (y-axis); significant taxa are highlighted. Statistical summaries and abundance tables detail W-values and normalized abundances across groups

Beta diversity metrics and ordination plots showed no clear group separation, likely due to high within-group variability and low-abundance taxa (data not shown). To identify differentially abundant taxa more reliably, we applied ANCOM, which compares each taxon relative to all others and reduces false discoveries (Fig. 3). When analyzing all sample types collectively, the genera *Enterococcus* and *Pseudomonas* exhibited statistically significant differences in abundance between patients with varying sepsis outcomes post-decannulation. While *Enterococcus* resulted more abundant in patients not developing sepsis, the opposite was true for *Pseudomonas* (Fig. 3a).

Focusing solely on cannula samples, three genera showed significant changes: *Enterococcus and Finegoldia were* significantly less abundant in patients who developed sepsis, and *Diaphorobacter*, showed a significantly higher abundance in these patients (Fig. 3b). Notably, *Diaphorobacter* was detected in the cannulas of all septic patients but was never identified in their skin or blood.

### Connecting bacteria on ECMO cannulas with bacteremia: combining culturing and sequencing

**Fig. 4:**
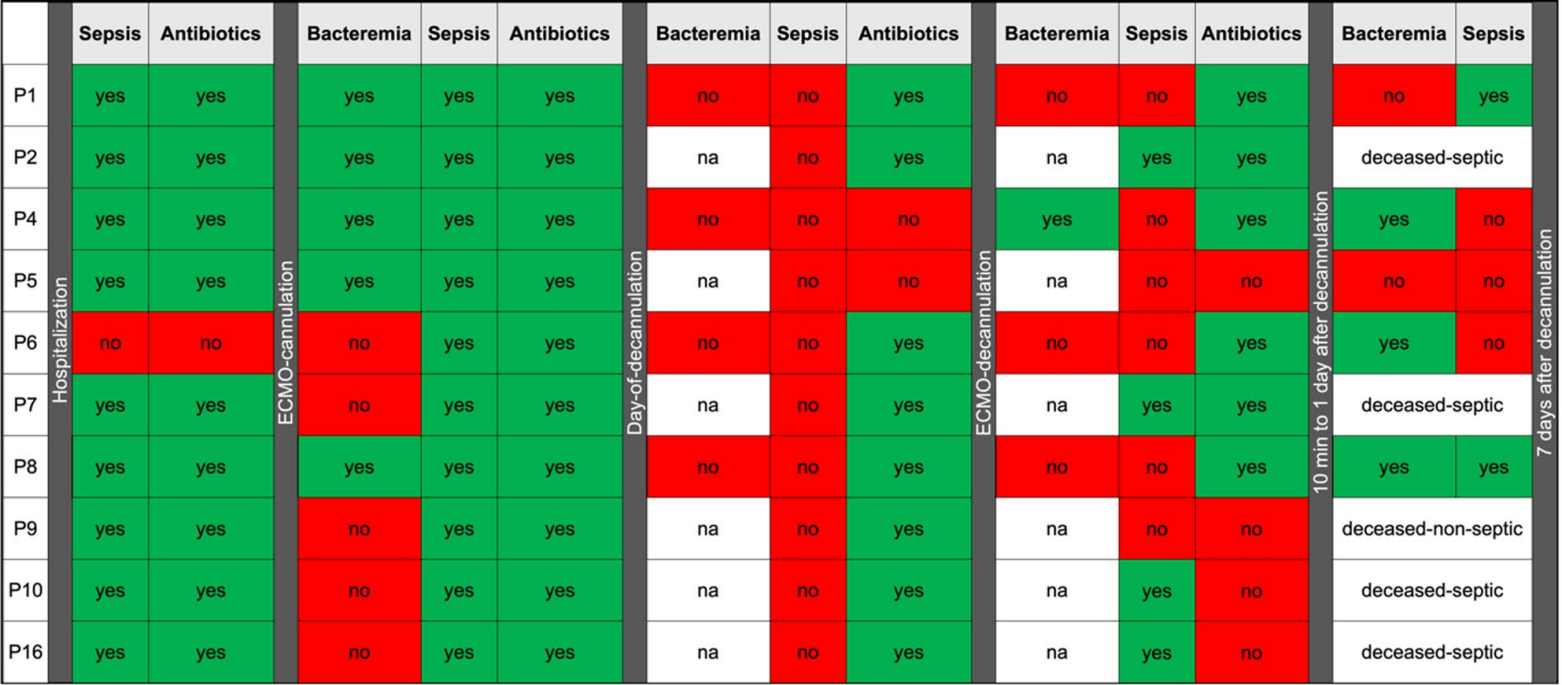
Overview of bacteremia (positive blood cultures), sepsis (SOFA scores), and antibiotic use before, during, and after ECMO in all patients. na: no blood cultures available

Bacteria detected on cannulas and in blood after decannulation were compared using microbiological and molecular methods. Figure 4 summarizes key events, including antibiotic treatment, sepsis and bacteremia for each patient. Supplementary Table 3 provides an overview of the microorganisms identified by culture and 16S rDNA in different sample types at various ECMO therapy stages.

Nine of ten patients developed sepsis prior to ECMO initiation and received antibiotic treatment. During ECMO therapy, all patients experienced sepsis; eight received antibiotics on the day of or during ECMO cannula removal. Five patients died during ECMO therapy and five survived. Two of five survivors developed sepsis within seven days after decannulation, with bacteremia detected by cultures only for one of them. Immediately after decannulation, only one patient developed a positive blood culture. All survivors showed inflammatory reactions on the day after decannulation, although SOFA score did not indicate sepsis.

**Fig. 5:**
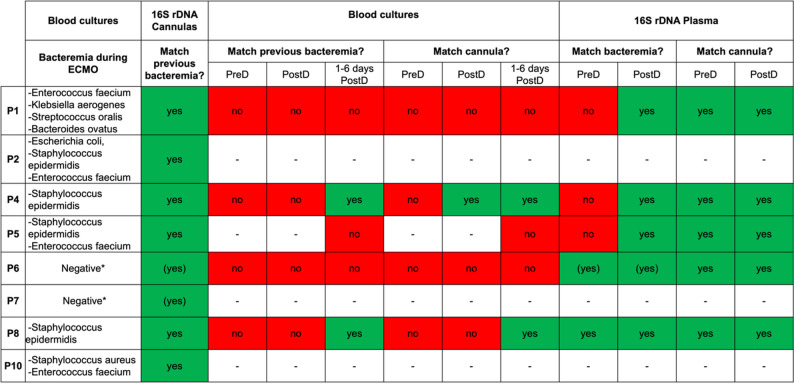
Comparison of bacteria detected during ECMO-related bacteremia with those found in cannulas and blood (by 16S rDNA and culture). Uncolored cells (–) indicate missing blood samples. (Yes) denotes cases where blood cultures were negative, but findings matched bacteria from central vascular catheters (CVC). Patient 9 had no bacteremia during ECMO therapy; data for patient 16 were unavailable

Figure 5 compares bacteria causing bacteremia during ECMO therapy with those detected in cannulas and blood. In all patients with positive blood cultures during the ECMO therapy, pathogens identified in cultures matched those detected on ECMO cannulas by 16S rDNA. This supports the notion that bacteria causing bacteremia during ECMO therapy can colonize cannulas and form biofilms. In four patients, the most abundant pathogens identified on the cannula were also present in high abundance in swabs from skin distal to and at the insertion-site (Supplementary Table 3), suggesting the skin as an additional source of cannula-colonizing bacteria.

Blood samples were only analyzed for the surviving patients. As shown in Fig. 4, none had ongoing bacteremia prior to decannulation. However, 3 out of 5 patients developed at least one positive blood culture within the seven days after ECMO therapy. Interestingly, except for one patient, all blood cultures were negative immediately after decannulation. However, 16S rDNA detected bacteria in the plasma of all surviving patients at this stage. These pathogens matched those from prior infections and those identified on the cannulas, supporting the notion that bacteria from the cannulas are reintroduced into the bloodstream during the decannulation. For two patients, positive vascular catheter cultures were observed despite negative blood cultures during ECMO therapy (marked with * in Fig. 5). The respective pathogens were also detected on the ECMO cannula by 16S rDNA, suggesting possible transmission onto the cannulas through undetected bacteremia.

## Discussion

This study developed a workflow combining culturing and 16S rDNA sequencing to investigate the colonization of ECMO cannulas and its clinical consequences.

A key finding is that 16S rDNA amplicon sequencing detected circulating bacteria missed by conventional blood cultures, often corresponding to pathogens from prior infections presumed eradicated by antibiotics. Our results further show: (1) microbes colonizing ECMO cannulas matched those causing bacteremia during ECMO therapy, indicating persistence of bloodstream pathogens in indwelling devices; (2) pathogens responsible for post-decannulation bacteremia matched those detected on cannulas, suggesting reintroduction into the bloodstream during decannulation; (3) bacterial diversity in plasma and insertion sites increased after decannulation, likely reflecting release of additional bacteria from cannulas; (4) higher bacterial diversity in cannulas was associated with an increased risk of subsequent sepsis; (5) non-septic patients exhibited higher *Enterococcus* abundance, whereas patients developing sepsis showed higher Pseudomonas abundance; and (6) 16S rDNA allowed early detection of bloodstream pathogens that evade conventional traditional culture but may later cause bacteremia. These findings may help inform future strategies for sepsis risk assessment following ECMO decannulation.

Previous studies link ECMO cannula biofilms to localized and systemic infections [19], with matching pathogens found on cannulas and in blood [9, 10, 35]. This suggests that biofilms on ECMO cannulas may play a key role in infection pathogenesis and serve as a conduit for systemic dissemination. Our study supports this concept, showing that bloodstream infections during ECMO therapy promote cannula colonization, even when the initial infection was considered resolved, as indicated by negative blood cultures. This suggests that pathogens may persist in the bloodstream at levels below the detection limit of conventional cultures, but detectable by 16S rDNA sequencing. Conversely, a recent study found no correlation between ECMO cannulas colonization and clinical outcomes [18].

A distinctive feature of our study is the integration of conventional culturing with 16S rDNA analysis, which provided evidence that ECMO decannulation can release bacteria from cannulas back into the bloodstream. In some cases, this was associated with sepsis or at least an inflammatory response detectable by clinical and laboratory parameters. Pathogens identified on the skin and at ECMO cannula insertion sites were also detected in plasma samples from some patients, underscoring the role of decannulation in facilitating bacterial translocation.

Our findings suggest that antibiotic prophylaxis during ECMO decannulation warrants critical re-evaluation. Current standard practice typically involves obtaining blood cultures and administering antibiotics based on suspected pathogens when new sepsis develops [36]. Given accumulating evidence that ECMO cannulas act as bacterial reservoirs [8, 9, 18, 19] and our finding that decannulation may reintroduce pathogens previously circulating into the bloodstream, the choice of empiric antibiotic therapy becomes crucial. In this context, it may be appropriate to consider using antibiotics effective against the patient’s prior biofilm-forming infections, especially if sepsis occurs post-decannulation. The role of antibiotic prophylaxis during ECMO treatment remains uncertain and requires further investigation [37, 38]. Any prophylactic strategy must be patient-specific and time-limited to minimize the risk of promoting antimicrobial resistance. Within this framework, a short, targeted prophylactic regimen administered immediately before decannulation, guided by the patient’s prior infection history, may merit further evaluation.

Developing early risk stratification systems predicting sepsis susceptibility is crucial, as timely treatment improves outcomes. Our study contributes to this objective in two ways. First, higher alpha diversity in cannulas of septic patients suggests bacterial richness may signal adverse outcomes. A previous study reported slightly higher alpha diversity on ECMO cannulas for non-bacteremic patients [19]. However, the limited sample sizes in both studies underscore the need for further research to clarify the relationship between microbial diversity on ECMO cannulas and sepsis risk. Second, not only bacterial richness but specific taxa affect outcomes: higher *Diaphorobacter* abundance in ECMO cannulas was associated with sepsis, while *Enterococcus* and *Finegoldia* were more abundant in cannulas of non-septic patients. Across all sample types, *Pseudomonas* was enriched in septic patients, while *Enterococcus was* predominant in non-septic patients. These findings suggest that these genera could serve as potential biomarkers for patient outcomes. Although the small-scale of our study limits definitive conclusions, the detection of such trends in a limited cohort highlights the remarkable potential of this approach for identifying ECMO risk stratification biomarkers.

Among genera linked to patient outcomes, *Finegoldia* is part of the normal flora of the skin and mucous membranes but can act as an opportunistic pathogen [39]. *Enterococcus*, another common commensal, forms biofilms and resists harsh conditions [40–42]. Antibiotic exposure may promote resistance in *Enterococcus*, increasing its pathogenic potential [40, 43]. Interestingly, in this study higher *Finegoldia* and *Enterococcus* abundance was associated with reduced sepsis risk, implying that these genera might promote protective microbiota shifts. *Pseudomonas*, containing known opportunistic pathogens has been previously associated with cannula-related infections [8, 35]. While *Diaphorobacter* hasn’t been linked to sepsis, related Comamonadaceae have been found in port-catheter-related infections [44]. These observations highlight the need for further research into the role of specific genera in ECMO-related sepsis.

This study has limitations, including the small sample size, which prevents the generalization of our findings but highlights important aspects warranting investigation in larger cohorts. Additionally, as a single-center study, the results may reflect a location-specific pathogen profile unique to this intensive care unit.

## Conclusions

Our findings corroborate that ECMO cannulas act as reservoirs for pathogens from previous infections, with decannulation facilitating bacterial re-entry into the bloodstream and contributing to post-decannulation sepsis.

These results differs from the findings of Kreitmeier et al. [18] who see no connection between bacteria in catheters and patient outcomes based on traditional culture assays combined with a molecular approach to quantify bacterial DNA, and extends the conclusions of Yu et al. [19] who correlate bacterial colonization of cannulas with poor patient outcomes. Both Yu et al. [19] and our study indicate that the composition of the cannula microbiome plays a key role.

It is the use of 16S rDNA analysis that supported unveiling the composition of the cannula microbiome and thus allowed correlating its composition with patient outcomes. While this technique does not offer the same resolution as full genome sequencing, its resolution is comparable to mass spectrometry with a speed and cost advantage over genome sequencing. In addition, no isolation or cultivation is required, this technique is applied directly to clinical samples.

Although 16S rDNA amplicon sequencing is not yet broadly implemented in routine clinical diagnostics, recent improvements in databases, analysis pipelines, and sequencing platforms have made rapid, low-cost identification of clinically relevant, well-characterized bacterial groups increasingly feasible [45–47]. When integrated into existing culture-based workflows, this method can assist in situations where culture is slow, inconclusive, or misses low-abundance organisms, as observed in our study. While it cannot provide antimicrobial resistance information and species-level resolution remains under active evaluation for many pathogens, 16S rDNA sequencing offers a practical way to monitor microbial community changes, support early recognition of pathogen re-emergence, and detect unexpected or overlooked taxa in complex infections. Employed in this complementary role, it can broaden and accelerate diagnostic insights without replacing standard clinical methods.

Our focus on post ECMO outcomes and the comparison with prior bacteremia led us to detect the fact that biofilms appear to shield pathogens during ECMO, allowing them to re-emerge even when they are no longer detectable by culture-based methods.

These data support re-evaluation of prophylactic strategies during ECMO decannulation and provide a basis for further studies to develop early sepsis risk stratification tools.

## Supplementary Information

Below is the link to the electronic supplementary material.

Supplementary Material 1

Supplementary Material 2

## Data Availability

The sequencing data generated in this study are available in the European Nucleotide Archive (ENA) under accession number PRJEB93957.

## Abbreviations

ECMO: Extracorporeal membrane oxygenation
VV: Venovenous
VA: Venoarterial
ARDS: Respiratory distress syndrome
16S rDNA: 16S ribosomal DNA
OTU: Operational taxonomic units
ASV: Amplicon sequence variants
ANCOM: Analysis of composition of microbiomes
