# Supplementary material for: Biofilm formation on venovenous ECMO cannulas can lead to re-introduction of pathogens during the decannulation process – a small-scale study reveals new insights when combining cultures and molecular results
Source: BMC Infect Dis. 2026 Feb 5;26:273. doi: 10.1186/s12879-026-12731-x (PMC12874949; doi:10.1186/s12879-026-12731-x)
Supplement: Supplementary file 2 — Supplementary Material 2 [file 12879_2026_12731_MOESM2_ESM.docx]

# **Supplementary Tables**:

Supplementary Table 1. Demographics and characteristics of study subjects.

| **Variables** | **living (n=5)** | **Dead (n=5)** | **All n=10** |
| --- | --- | --- | --- |
| Characteristics |  |  |  |
| Gender (M/F) | 3/2 | 4/1 | 7/3 |
| Age | 43 [30-66] | 55 [43-63] | 50 [39-65] |
| BMI | 29 [26-30] | 28 [25-47] | 29 [27-32] |
| Pre-existing conditions |  |  |  |
| diseases | 3 [1-6] | 4 [2-5] | 3 [1-5] |
| Lung diseases (yes/no) | 2/3 | 2/3 | 4/6 |
| Immunosuppression (yes/no) | 1/4 | 2/3 | 3/7 |
| Duration of |  |  |  |
| Hospitalization pre-intubation (days) | 3 [0-26] | 1 [0-13] | 2 [0-12] |
| Intubation pre-ECMO (days) | 0 [0-1] | 1 [0-4] | 1 [0-2] |
| ECMO therapy (days) | 30 [16-39] | 15 [7-39] | 29 [8-38] |
| ICU stay (days) | 34 [22-50] | 18 [10-39] | 32 [13-40] |
| Infections |  |  |  |
| Sepsis pre ECMO therapy (yes/no) | 4/1 | 5/0 | 9/1 |
| Sepsis during ECMO therapy (yes/no) | 5/0 | 5/0 | 10/0 |
| Sepsis after ECMO therapy (yes/no) | 3/2 | n.a. | 3/2 |
| Pneumonia (yes/no) | 4/1 | 5/0 | 9/1 |
| Antibiotics pre ECMO therapy (yes/no) | 4/1 | 5/0 | 9/1 |
| Antibiotics during ECMO therapy (yes/no) | 5/0 | 5/0 | 10/0 |
| Antibiotics during ECMO cannula removal (yes/no) | 3/2 | 5/0 | 8/2 |
| Antibiotics after ECMO therapy (yes/no) | 4/1 | n.a. | 4/1 |
| Microbiological evidence |  |  |  |
| Bacterial detection pre ECMO therapy(yes/no) | 1/4 | 1/4 | 2/8 |
| Bacterial detection during ECMO therapy (yes/no) | 4/1 | 4/1 | 8/2 |
| Bacterial detection after ECMO therapy (yes/no) | 3/2 | n.a. | 3/2 |
| Abbreviations: BMI: body-mass index; ECMO: extra corporeal membrane oxygenation; ICU: intensive care unit; n.a.: not applicable. | | | |

Supplementary Table 2: Pairwise comparisons (Kruskal-Wallis test) of alpha diversity between samples using the Chao1 index and computing samples from all sites (a) or only from cannulas (b).

| a) All Sites | Group 1 | Group 2 | H | p-value |
| --- | --- | --- | --- | --- |
| Site and stage from decannulation | plasma-after | plasma-before | 0.71 | 0.400 |
|  | plasma-after | cannula | 3.65 | 0.056 |
|  | plasma-after | insertion-site-after | 5.18 | 0.023 |
|  | plasma-after | insertion-site-before | 1.07 | 0.300 |
|  | plasma-after | skin-distal | 4.69 | 0.030 |
|  | plasma-before | cannula | 4.53 | 0.094 |
|  | plasma-before | insertion-site-after | 6.24 | 0.057 |
|  | plasma-before | insertion-site-before | 2.89 | 0.089 |
|  | plasma-before | skin-distal | 8.14 | 0.004 |
|  | cannula | insertion-site-after | 1.39 | 0.238 |
|  | cannula | insertion-site-before | 0.73 | 0.393 |
|  | cannula | skin-distal | 2.47 | 0.116 |
|  | insertion-site-after | insertion-site-before | 2.75 | 0.097 |
|  | insertion-site-after | skin-distal | 0.08 | 0.766 |
|  | insertion-site-before | skin-distal | 2.50 | 0.114 |
| b) Cannulas |  |  |  |  |
| Bacteremia | na (deceased) | no | 3.85 | 0.074 |
|  | na (deceased) | yes | 8.41 | 0.011 |
|  | no | yes | 277 | 0.096 |
| Sepsis (Sofa) | na (deceased) | no | 3.47 | 0.062 |
|  | na (deceased) | yes | 11.36 | 0.002 |
|  | no | yes | 5.49 | 0.029 |

Supplementary Table 3. Bacteria identified in patient samples by culture and 16S rDNA amplicon sequencing during ECMO therapy, immediately before and after the decannulation, and during the 7-days after decannulation. The 16S results for each patient were grouped according to the sample sites into the following categories: skin-distal, corresponding to the patient’s skin swabs away from the cannula insertion site; insertion site before and after decannulation (with after including the skin swabs at the insertion site and the compresses applied to the insertion site); catheter; and plasma before and after decannulation, as described in the methods section.

(attached-Excel file)
